# Crosslinking Constraints and Computational Models as Complementary Tools in Modeling the Extracellular Domain of the Glycine Receptor

**DOI:** 10.1371/journal.pone.0102571

**Published:** 2014-07-15

**Authors:** Zhenyu Liu, Agnieszka Szarecka, Michael Yonkunas, Kirill Speranskiy, Maria Kurnikova, Michael Cascio

**Affiliations:** 1 Center for Neuroscience, University of Pittsburgh, Pittsburgh, Pennsylvania, United States of America; 2 Department of Chemistry, Carnegie Mellon University, Pittsburgh, Pennsylvania, United States of America; 3 Department of Cell and Molecular Biology, Grand Valley State University, Allendale, Michigan, United States of America; 4 Department of Chemistry and Biochemistry, Duquesne University, Pittsburgh, Pennsylvania, United States of America; University of Pittsburgh, United States of America

## Abstract

The glycine receptor (GlyR), a member of the pentameric ligand-gated ion channel superfamily, is the major inhibitory neurotransmitter-gated receptor in the spinal cord and brainstem. In these receptors, the extracellular domain binds agonists, antagonists and various other modulatory ligands that act allosterically to modulate receptor function. The structures of homologous receptors and binding proteins provide templates for modeling of the ligand-binding domain of GlyR, but limitations in sequence homology and structure resolution impact on modeling studies. The determination of distance constraints via chemical crosslinking studies coupled with mass spectrometry can provide additional structural information to aid in model refinement, however it is critical to be able to distinguish between intra- and inter-subunit constraints. In this report we model the structure of GlyBP, a structural and functional homolog of the extracellular domain of human homomeric α1 GlyR. We then show that intra- and intersubunit Lys-Lys crosslinks in trypsinized samples of purified monomeric and oligomeric protein bands from SDS-polyacrylamide gels may be identified and differentiated by MALDI-TOF MS studies of limited resolution. Thus, broadly available MS platforms are capable of providing distance constraints that may be utilized in characterizing large complexes that may be less amenable to NMR and crystallographic studies. Systematic studies of state-dependent chemical crosslinking and mass spectrometric identification of crosslinked sites has the potential to complement computational modeling efforts by providing constraints that can validate and refine allosteric models.

## Introduction

Anionic–selective glycine receptors (GlyRs^5^) play critical roles in fast neuronal communication and in neural development. This neurotransmitter-gated channel is a member of the pentameric ligand-gated channel (pLGIC) superfamily of receptors (also referred to as Cys-loop receptor due to a conserved disulfide loop in each subunit) that also include GABA receptors (GABARs), nicotinic acetylcholine receptors (nAChRs), and serotonin receptors (5HT_3_Rs). Each pLGIC subunit has a large N-terminal extracellular domain (ECD), with a ligand-binding site located at the interface between adjacent subunits [1], [2]. Each of the five subunits has four membrane-spanning segments, with the second segment lining its central gated pore. Upon binding of ligands in the ECD, complex allosteric changes in conformation result in transient opening of a distant gate in the central pore in the transmembrane domain of the receptor and subsequent desensitization [3]. Thus, conformational changes driven by the free energy changes upon ligand binding in the ECD are structurally coupled to channel gating events, presumably via associations/contacts between this domain and the transmembrane domain (TMD, which include the loops connecting transmembrane segments) [4]–[7]. This functional coupling of the ECD and TMD in GlyR is supported by the presence of point mutations in the interfacial loops linking these two domains in GlyR sequences of some individuals with hyperekplexia, a neurological disease characterized by an excessive startle response wherein channel gating is effectively uncoupled from ligand binding [8].

The available experimental structures of pLGICs include a cryo-electron microscopy structure of *Torpedo* nAChR at 4 Å resolution [9], crystal structures of a *C. elegans* glutamate-gated Cl channel [10] and homologous bacterial pLGICs [11]–[15], an X-ray structure of a monomeric form of an ECD of nAChR [16], and X-ray structures of acetylcholine binding protein (AChBP; a homolog of nAChR ECD) bound to a variety of ligands [17]–[23]. However, these structures have not provided any direct evidence on the molecular details involved in interactions of the receptor with its cognate ligands and any subsequent allosteric effects of ligand binding, nor do they provide any direct information regarding the structure of the ECD of GlyR. In previous studies, we created and tested homology models of the ECD of GlyR based on AChBP and nAChR-ECD as templates [24]. Although AChBP has limited sequence identity to the ECD of Cys-loop receptors, biochemical and high-resolution structural studies have shown that it is a good template for modeling of these domains [25], [26]. Our initial three-dimensional structure of the GlyR-ECD pentamer was constructed using comparative modeling and further refined using equilibrium molecular dynamics (MD) simulations resulting in the model of the fully hydrated pentamer that was stable for the entire 5 ns simulation. The relative structural drift measured as the root mean square deviation (RMSD) of C_α_ atoms from the initial structure was <3.5 Å, a relatively small value considering that the starting structure was a homology model. This stability indicates also that AChBP, despite low sequence identity with pLGICs, is a very good template for its ligand binding ECD. The structure of the binding pockets in the homology model was also consistent with published biochemical studies. While homology models provide insight into protein structure and function, these models carry uncertainties with respect to molecular details at high resolution, particularly in regions where sequence variability is highest. In the case of the ECD of Cys-loop receptors, these variable regions include the ligand binding pockets and the subunit interfaces as these areas define the unique features of receptor subtypes, such as ligand specificity and stoichiometry upon assembling. Thus an experimental approach (such as chemical crosslinking coupled with mass spectrometry (MS), as utilized in this study) that provides direct and indirect structural information about the protein, employed in combination with computational modeling has the potential to resolve some of these uncertainties. In addition, the integration of modeling and systematic experimental measurements has the potential to identify subtle conformational changes associated with receptor gating and desensitization.

Chemical crosslinkers can function as molecular rulers as covalent inter- and intra-subunit bonds provide distance constraints that may be used to refine tertiary and quaternary structures of proteins [27]–[30]. Chemical crosslinking/MS analyses have been used to provide structural information for modeling membrane proteins and larger macromolecular complexes and are capable of providing critically needed information regarding local conformational dynamics [31]–[37]. With respect to pLGICs, there is a rich history of using crosslinking studies to elucidate receptor structure and function [38]–[47]. In this study, we used a soluble crosslinking agent that does not require mutagenesis to introduce potential crosslinkable moieties but instead reacts with accessible endogenous Lys residues. Dimethylsuberimidate (DMS), a homo-bifunctional amine-reactive reagent, specifically crosslinks primary amine groups (*i.e*., ε-amino groups of lysine residues) and has been widely used to map low-resolution protein structures [48]–[50]. Due to its high sensitivity, mass accuracy, and high throughput, MS has the unique ability to provide large amounts of structural data in systems (e.g. low abundance proteins and/or very large complexes) not easily handled by conventional techniques [28], [35], [51], [52]. The coupling of crosslinking studies with subsequent mass spectrometric (MS) analyses allows one to sensitively identify introduced crosslinks, even if reaction products are in low abundance.

pLGICs, similar to other integral membrane proteins, provide a hurdle to characterization as they are typically expressed at fairly low abundance and are embedded in the lipid bilayer which provides a hindrance for many biophysical techniques typically used to characterize protein structure. With respect to the latter concern, pLGICs may be solubilized by detergents, but this substitution of a membrane-mimetic environment for the natural bilayer still makes characterization difficult and may affect native structure. In order to focus on the ECD of GlyR and to eliminate the need for solubilizing agents and ease difficulties presented by the particulate nature of the bilayer, we have chosen to conduct experiments on a soluble, truncated form of GlyR. We have previously expressed and purified a pentameric glycine-binding protein (GlyBP) that was shown to be a structural and functional homolog of its corresponding ligand binding domain in full-length GlyR [53]. Examination of GlyBP can thus be expected to provide important insights into the tertiary and quaternary contacts in pLGICs, particularly in GlyR, whose experimental structure is poorly resolved. Herein we report our efforts to obtain structural information provided by crosslinking/mass spectrometry (MS) studies of GlyBP to identify and differentiate intra- and intersubunit crosslinks. Our computational GlyBP model provides the template wherein we evaluate whether we can differentiate these two classes of crosslinks. By comparing the assigned crosslinks derived from MALDI-TOF (Matrix-Assisted Laser Desorption Ionization – Time Of Flight) MS studies of monomeric or oligomeric bands after SDS-PAGE, assignments were made identifying inter-subunit crosslinks and intra-subunit crosslinks via identification of parent mass ions. All assigned crosslinks were consistent with our computational model, providing confidence that we can distinguish inter- and intra-subunit crosslinks in our homomeric protein. Thus crosslinking/MS studies are potentially capable of providing a network of intra- and intersubunit constraints to critically evaluate and refine structural models of GlyR and other pLGICs.

## Experimental Procedures

### Expression and Purification of GlyBP

**Figure 1:**
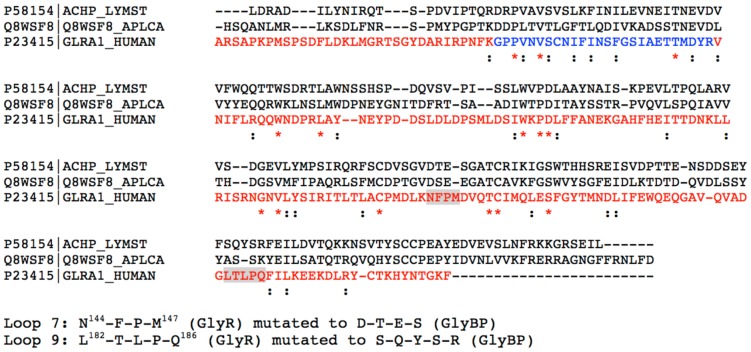
Coverage map and CLUSTAL 2.1 multiple sequence alignment (after manual adjustments described in [24]) of L. stagnalis. A. californica, and the extracellular domain (ECD) of human glycine receptor alpha1 subunit. Loops 7 and 9 of GlyR ECD have been mutated to obtain GlyBP (grey highlights on the alignment). Sequence highlighted in red cumulatively marks peptides whose mass ions are detected in control studies. As described in the text, tryptic fingerprinting of GlyBP gel slices typically resulted in 55–80% coverage.

GlyBP was expressed and purified as described previously [53]. In order to produce this soluble form of the ECD of GlyR several residues in two loops were mutated to substitute relatively hydrophilic sequences found to AChBP in the place of the more hydrophobic loops found in α1 subunits at the ECD-TMD interface (**Fig. 1**: multiple sequence alignment with loop 7 N144-F-P-M147 to D-T-E-S and loop 9 L182-T-L-P-Q186 to S-Q-Y-S-R highlighted). Briefly, *Spodoptera frugiperda* (Sf9) cells (Invitrogen) were grown in Grace’s Insect Medium supplemented with 10% fetal bovine serum (FBS) and 100 U/ml penicillin/100 µg/ml streptomycin at 28°C as suspension cultures in spinner flasks under constant rotation (120 rpm). Sf9 cells were infected with virus encoding GlyBP at MOI > 5 and harvested 4 days post-infection. Harvested Sf9 cells were gently pelleted by centrifugation at 1000×g for 10 min. Cells were washed three times with ice-cold PBS and resuspended on ice for 1 h in hypotonic solution (5 mM Tris (pH 8.0), 5 mM EDTA, 5 mM EGTA, 10 mM dithiothreitol, and an anti-proteolytic cocktail containing 1.6 µu/ml aprotinin, 100 µM phenylmethylsulfonyl fluoride, 1 mM benzamidine and 100 µM Benzethonium chloride). Cells (jacketed in an ice bath) were lysed by probe sonication using a microtip (8×15 sec, using a 50% cycle). Disrupted cells were centrifuged at 100,000 g for 1 h. Pellets were resuspended at 4°C overnight in solubilization buffer (25 mM KP*_i_* (pH 7.4), 1% digitonin, 0.1% deoxycholate, 0.5 mg/ml Egg PC, 500 mM KCl, 5 mM EDTA, 5 mM EGTA, 10 mM dithiothreitol, and our anti-proteolytic cocktail). Samples were then centrifuged at 100,000×g and the solubilized supernatant added to 2-aminostrychnine agarose matrix at 4°C overnight with gentle agitation. The agarose was washed three times with excess wash buffer (solubilization buffer with digitonin reduced to 0.1%), and then eluted for 2 days with solubilization buffer containing 1.5 mM 2-aminostrychnine. The eluate was dialyzed against 100 mM KCl/25 mM KP*_i_* (pH 7.4) for 2 h, 20 mM KCl/25 mM KP*_i_* (pH 7.4) for another 2 h and then dialyzed against 25 mM KP*_i_* (pH 7.4) overnight. After centrifugation at 100,000×g, the pellet (membrane associated form) was resuspended in 25 mM KP*_i_* buffer with a final protein-lipid ratio of ∼1∶200 (mol:mol) and the supernatant (aqueous form) was concentrated in an Amicon Ultra-4 centrifugal filter device with a 10 KDa cutoff. Protein concentrations were determined by modified Lowry assay [54]. For SDS-PAGE, protein samples were treated with SDS-PAGE sample buffer containing 2% SDS and heated for 5 min at 95°C. Proteins were separated by 10% SDS-PAGE and transferred to nitrocellulose. Western immunoblots were developed with monoclonal anti-mouse antibodies against GlyR and horseradish peroxidase (HRP)-conjugated secondary antibody using standard protocols.

### Homology modeling and Molecular Dynamics refinement of GlyBP model

The previously published structure of the ECD of wild-type GlyR [24] was used to generate a model of GlyBP. Briefly, the GlyR sequence was aligned with those of *L. stagnalis* AChBP (20% sequence identity) and *A. californica* AChBP (13% sequence identity) using Clustal W [55] as shown in **Fig. 1**. The alignment was manually adjusted as described in detail in Speranskiy *et al.* [24]. The program MODELLER was used to generate a model of the GlyBP pentamer [56]. All five subunits of the pentamer were modeled simultaneously using five-fold symmetry. The MODELLER’s variable target function method (VTFM) and molecular dynamics simulated annealing generated 15 initially randomized models. The quality of these models was characterized in terms of Z-scores using the WHAT_IF program [57]. Z-scores are standardized statistically-derived structure quality assessment scales that include packing quality, Ramachandran plot appearance, chi-1/chi-2 rotamer normality, and backbone conformation.

The highest quality model was selected for further refinement using Molecular Dynamics simulations carried out in AMBER simulation package ([58] and following the protocol as described in in previous studies [24]. Briefly, the protein structure was solvated in explicit atomistic water (the TIP3P water model was used as implemented in AMBER). After short minimization with steepest descent algorithm, equilibration MD simulations (a single trajectory) were carried out starting at low temperature of 10 K and heating to the constant temperature 300 K. The initial simulations were carried out at constant pressure of 1 atm. Hydrogen bonds were constrained using SHAKE algorithm, the integration time-step was 2 fs, Berendsen thermostat was used to maintain constant temperature, the long range electrostatics was evaluated using Particle Mesh Ewald method with non-bonded interaction cut off at 10Å, all as implemented in AMBER. After equilibration a production trajectory was simulated for 5 ns, at constant temperature 300 K and constant volume. The simulations produced a stable GlyR-ECD pentamer structure, whose coordinates were used to initiate the model of GlyBP (by residue substitutions as shown in **Fig. 1**). The GlyBP model was then further simulated in a similar fashion as described above to ensure proper equilibration and simulation of the mutant loops. The average lysine inter-residue distances were calculated based on the last 2 ns of the trajectory. Trajectory analysis and all molecular images were drawn using the Visual Molecular Dynamics (VMD), version 1.8.6 [59].

### Chemical crosslinking

Aqueous forms of GlyBP were expressed and purified as described previously [53] and incubated with various concentrations of DMS (0.2 to 2 mg/ml) at room temperature for 1 hr. Protein samples were also incubated at RT without addition of DMS as controls. Reactions were quenched with addition of Tris buffer at a final concentration of 50 mM. Crosslinked or non-crosslinked proteins were separated on SDS-PAGE. After electrophoresis, gel bands were visualized by silver stained using standard protocols. After silver staining the gel was destained with ultrapure water and bands of interest were excised and transferred into microcentrifuge tubes. Analogous control pieces of gel of the same approximate size from a gel lane without loaded protein were similarly excised and processed.

### In-gel trypsin digestion

Before trypsin digestion, cysteine residues were reduced and alkylated with iodoacetamide. Briefly, the gel pieces were incubated with 100% acetonitrile, and then 10 mM DTT, 100 mM TrisHCl, pH 8.5 at 55°C for 1 h. After two washes with 100 mM TrisHCl, pH 8.5, the gel pieces were incubated with 15 mM iodoacetamide in 100 mM TrisHCl, pH 8.5 for 1 h in dark at room temperature. Gel pieces were washed with 50∶50 methanol: 50 mM ammonium bicarbonate twice for 30 min with gentle agitation. The gel plugs were dehydrated by adding 50 µl acetonitrile. After the gel plugs turned whitish, acetonitrile was removed and gel slices were dried in a SpeedVac for approximately 15 min. 10 µl of trypsin in solution (20 µg/ml of porcine trypsin in 20 mM ammonium bicarbonate) was added to each sample and samples were put on ice for 15 min and incubated overnight at 37°C. Digested peptides extracted into solution were transferred to a new tube. To further extract the tryptic fragments, gel plugs were incubated for 30 min in 60 ul of 1% TFA in 50∶50 acetonitrile: H_2_O with gentle agitation. The liquid was extracted and saved. Gel pieces were washed twice more, and the rinses were combined with previous extracts and dried in a SpeedVac without heating.

### Matrix-assisted laser desorption/ionization time-of-flight mass spectrometry (MALDI-TOF MS)

Prior to sample spotting, protein samples were purified and concentrated using C18 Ziptips (Millipore). The Ziptip was pre-wet by 10 µl of 50% acetonitrile in Milli-Q water, equilibrated with 10 µl of 0.1% TFA in Milli-Q water. The sample was drawn up into Ziptip and pipetted up and down 5–6 times. The Ziptip was then washed twice with 10 µl of 0.1% TFA to remove contaminants. The peptides were eluted with 3 µl of 50% acetonitrile/0.1% TFA in Milli-Q water into a labeled clean vial. Those samples were used for direct spotting for MALDI-TOF analysis. Samples were prepared by the dried-droplet method [60]. Briefly, in a clean microcentrifuge tube, 0.5 µl of each protein sample cleaned by C18 Ziptip was mixed with the same volume of 10 mg/ml of α–cyano-4-hydroxycinnaic acid (CHCA) (Applied Biosystems, Foster City, CA) in 50% acetonitrile/0.1% TFA by vortexing. The mixture of sample/matrix was deposited onto a welled gold sample plate. Droplets were air-dried at room temperature. A standard mixture including des-Arg^1^-Bradykinin ([M + H]^+^_mono = _904.47), Angiotensin ([M + H]^+^_mono = _1296.69), Glu^1^-Fibrinopeptide B ([M + H]^+^_mono = _1570.68), ACTH (1–17 clip) ([M + H]^+^_mono = _2093.09), ACTH (18–39 clip) ([M + H]^+^_mono = _2465.20) and ACTH (7–38 clip) ([M + H]^+^_mono = _3657.93) was used as an external calibrant. MALDI-TOF MS was performed on a Voyager DE Pro Biospectrometry Workstation equipped with a nitrogen laser (337 nm) at the Genomics and Proteomics Core Laboratories at the University of Pittsburgh. The instrument was run in positive ionization mode and measurements were conducted in reflector mode.

### Data analysis

MALDI-TOF mass spectra were analyzed by the Data Explorer software version 4.5 (Applied Biosystems). Crosslinking products were identified using the General Protein Mass Analysis for Windows (GPMAW, version 6.0) (Lighthouse Data, Odense, Denmark) and the Automated Spectrum Assignment Program (ASAP) developed at the University of California at San Francisco [61].

## Results

Members of the pLGIC family are homologous and high resolution structures of any member of this family is expected to provide good templates for modeling other pLGIC members and aid in our understanding of the functioning of these ion channels. In a previous report we described a homology model of the ECD of GlyR that proved remarkably stable during simulation, lending itself to further studies of dynamics of ligand binding and promising insight into the structure and behavior of the receptor [24]. However, while structural templates of pLGICs, or domains thereof, are available and are adequate for constructing initial models, these models need to be validated against a range of experimental data before they can be regarded as reliably representing the structure of the protein. The determination of systematically introduced constraints via crosslinking studies coupled with MS identification has the potential to provide essential experimental data to allow validation and/or refinement of these computational models (27–37). In order to provide proof-of-concept data to show the ability of MS to identify intra and inter-subunit crosslinks and provide the distance constraints that may be used to validate our models, we undertook crosslinking/MS studies to determine if we can distinguish intra- and intersubunit crosslinks upon treatment with a DMS, a lysine-specific crosslinking agent. We have chosen to conduct these studies using one-dimensional MALDI-TOF with fairly modest error tolerance (<50 ppm) to indicate that broadly available MS platforms are sufficient to identify crosslinked peptides and may be utilized in characterizing large protein complexes. These studies also provide initial distance constraints as they indicate that the two identified sites are sufficiently close enough to form a covalent crosslink.

### Homology model of GlyBP and MD simulations

**Figure 2:**
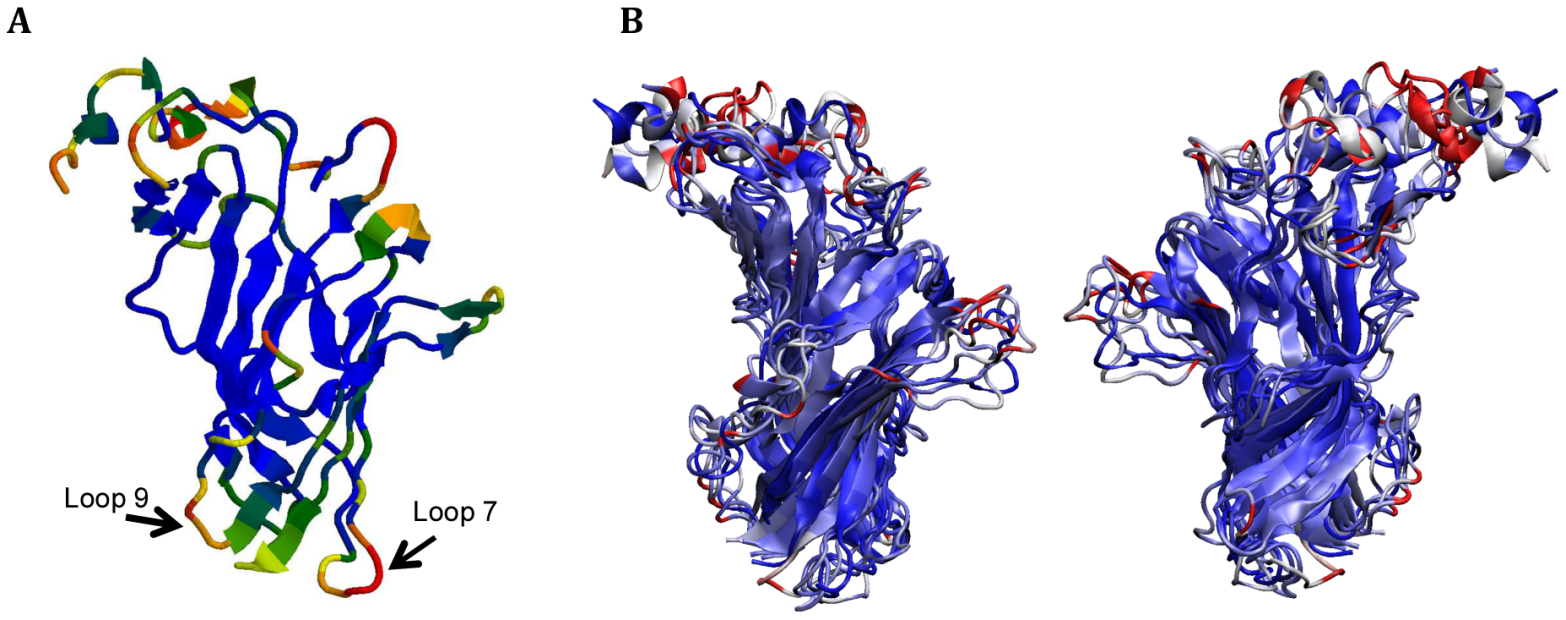
Model of GlyBP. A) Structure of a single GlyBP subunit is shown in a ribbon representation. Colors represent mobility of individual residues in MD simulations. Mobility is measured by root mean squared fluctuations (RMSF) with respect to an average structure obtained in a steady-state dynamics. The coloring scheme is as follows: RMSF <0.8 Å - blue, RMSF range 0.8–1.3 Å - green, 1.3–1.5 Å –yellow, 1.5–1.8 Å – orange, RMSF> 1.8 Å - red. B) Conformational diversity of subunits within the GlyBP pentamer is shown by a structural superposition of average monomer structures (last 2 ns of the trajectory) color coded by root mean squared deviation between subunits. Front (outer side) and back (inner side) are shown in the left and right panels, respectively.

The homology model of GlyBP was used to perform all-atom simulations of the GlyBP pentamer in water. The protein complex was stable over several nanoseconds of simulations and its fluctuations (calculated for the entire pentamer) did not exceed 3.5 Å compared to the initial model. This is consistent with our previous work where 5 ns simulations of the wild type extracellular domain of GlyR also revealed a stable structure with the structural drift of about 3.3 Å [24]. The difference between GlyR and GlyBP structures lies in several loops located on the surface proximal to the membrane in GlyR. In GlyR these loops are located at the interface between the ligand binding and transmembrane domains of the receptor and thus interact with the hydrophobic environment of the membrane and the transmembrane domain. In the GlyBP protein several hydrophobic residues in these loops have been mutated to the hydrophilic ones and the loops are fully exposed to water. Loops 7 and 9, which are implicated in extracellular-transmembrane communication, show increased relative mobility in simulations of the GlyBP model (**Fig. 2**) likely due to their more hydrophilic nature imparted by the substitutions. The Cα RMS fluctuations calculated over the last 2 ns and averaged for all subunits are mapped onto the GlyBP structure in **Fig. 2A**. **Fig. 2B** demonstrates the level of conformational diversity among the subunits (color coded by single subunit RMSD, inner and outer side shown on separate panels). Consistently, the N-terminal helix and the loop regions show the greatest conformational variation. In addition, **Fig. S1** shows the Cα RMSF profiles for individual subunits and the average Cα RMSF profile (thick red line).

### Chemical Crosslinking

**Figure 3:**
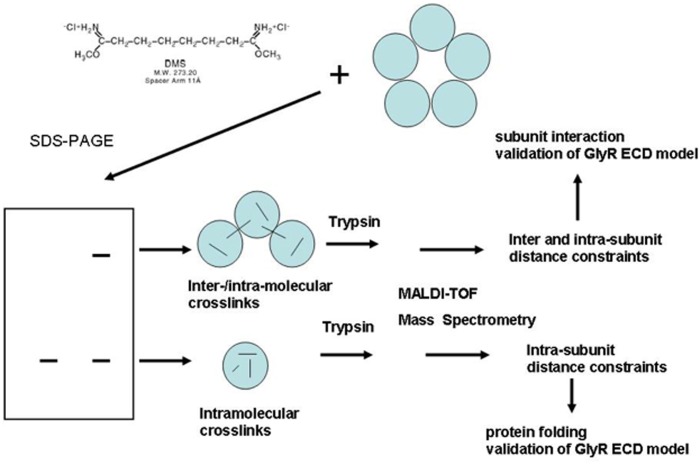
Overview of experimental strategy used in MS/GlyBP modeling studies.

A summary of the experimental design is schematized in **Fig. 3**. In GlyBP, there are a total 11 lysine residues which are potentially targeted by the amine-specific crosslinker DMS. In the absence of crosslinker, all lysine-containing peptides were identified and protein coverage of 55–80% of the entire sequence was obtained. Cumulatively, all tryptic fragments except for one were identified; in multiple runs we consistently were unable to identify the relatively large tryptic fragment [G^34^-R^59^] that encompass the N^38^ glycosylation site (GlyBP sequence and coverage map is shown in **Fig. 1** with the G^34^-R^59^ fragment marked blue and MS coverage shown in red). Crosslinking reactions were conducted using low micromolar concentrations of GlyBP to reduce crosslinks generated between oligomers. Thus all Lys-Lys crosslinking occurs within a single subunit or across subunit interfaces. To distinguish intra- and inter-subunit crosslinks, purified GlyBP was subjected to SDS-PAGE after crosslinking, separating lower- and higher-molecular weight bands corresponding to monomeric and oligomeric GlyBP, respectively. In the lower-molecular weight band, any crosslink must be intra-molecular, whereas in the higher-molecular weight bands, both intra- or inter-molecular crosslinks may exist. Lysine crosslinks were then identified by mass spectrometric fingerprinting studies of extracted tryptic peptides from the respective gel pieces. We hypothesized that any unique crosslinks identified solely in higher-order GlyBP oligomeric bands on SDS-PAGE may be assigned as inter-subunit Lys-Lys crosslinks. In the following sections, MS-identified crosslinks are mapped onto our computational model of GlyBP are evaluated to test this hypothesis.

### Identification of intrasubunit chemical crosslinks in GlyBP by MALDI-TOF MS

**Figure 4:**
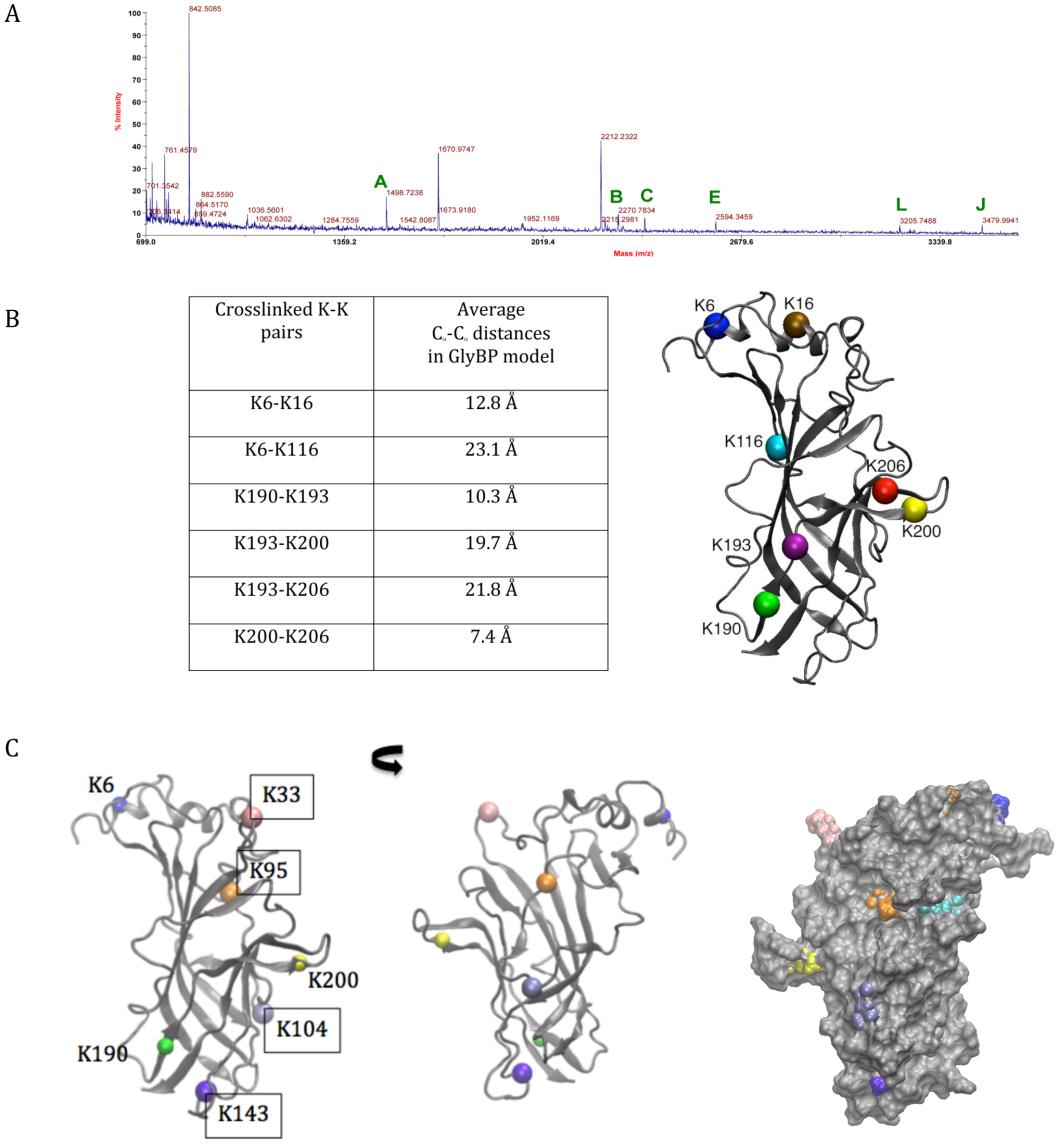
Intramolecular crosslinks observed in GlyBP by MALDI-TOF MS analysis after crosslinking with DMS. A) Representative mass spectrum of tryptic digest of excised monomeric GlyBP band. Mass peaks assigned as crosslinked peptides are labeled and further identified in **Table 1**. B) Average C_α_-C_α_ Lys-Lys distances measured along the MD trajectory of assigned crosslinks are provided in the panel below. The calculation of distances is averaged over all 5 subunits/interfaces over the 2 ns long MD trajectory. The positions of the Lys residues in the modeled GlyBP (for simplicity, only the monomer is represented) are shown. The protein structure is shown in grey color in cartoon representation and C_α_ atoms of Lys residues are shown as colored spheres. C) The positions of Lys residues in the model of GlyBP for which we did not observe crosslinking (residue numbers in boxes): the panel shows the outer surface and the inner surfaces of a subunit - the latter also represented by space-filled model.

Masses of tryptic peptides from crosslinked GlyBP were assigned from the mass spectra using ASAP and GPMAW. In the MALDI spectra, most peaks observed in the absence of DMS were also obtained in comparative studies conducted in the presence of the crosslinker (data not shown), indicating that modification of GlyBP by chemical crosslinking did not significantly interfere with tryptic digestion and subsequent MS studies. DMS-modified lysines are not targeted by trypsin as this chemical modification of the Lys sidechain eliminates its susceptibility to trypsinolysis. A representative MS spectrum of DMS-treated GlyBP is shown in **Fig. 4A**. Twelve unique mass ions that were present only in crosslinked samples were identified as K-K linked peptides and are listed in **Table 1**. These crosslinked peptides could be divided into two classes: those containing crosslinked K-K pairs within a single tryptic peptide and those with K-K crosslinked pairs between distinct tryptic peptides. In the former case, K190–K193 and K200–K206 crosslinks were observed within the peptides 187–196 and 197–213 respectively. These crosslinks are not unexpected given the close proximity of the lysine pairs in the primary sequence.

**Table 1:** Intramolecular crosslinks identified by mass spectrometric studies of monomeric GlyBP bands.

K-K crosslinks	Assigned peptide(s)	Theoretical m/z	Observed m/z	ΔMass, ppm*	N *	Peak*
190–193	187–196	1498.795	1498.714	−45	9	A
193–200	191–196, 197–206	2268.040	2268.133	−41	7	B
200–206	197–213	2357.048	2357.009	−16	6	C
N–6/16	1–2, 3–20	2430.195	2430.245	−20	7	D
193–206	191–196, 201–213	2593.214	2593.122	36	9	E
193–200	191–196, 194–206	2652.252	2652.312	−23	9	F
193–206	191–200, 201–213	3145.452	3145.393	19	6	G
190/193–200	187–196, 194–206	3153.584	3153.454	41	7	H
193–200	191–200, 194–206	3204.489	3204.330	50	9	I
6–116	3–16, 105–119	3478.711	3478.618	27	6	J
190/193–200/206	187–196, 197–213	3646.783	3646.624	44	6	K
N/6–116	1–16, 105–119	3705.849	3705.769	22	8	L

*the ΔMass is the maximum observed ppm difference between theoretical and observed m/z over N, the number of times this m/z peak was observed in 10 independent experiments. See **Fig. 4** for corresponding assigned peak in representative MALDI-TOF spectrum.

Considering the flexibility of Lys residues, it is expected that DMS can cross-link two lysine residues with C_α_- C_α_ distance up to 24 Å (the arm length of DMS plus two times the length of lysine side chain, which is about 6.5 Å) [61]. In our GlyBP homology model, K6 and K116 were assigned to the N-terminal α–helix and β5 respectively, as shown in **Fig. 4B**. The top portions of the subunits, including the short α–helix, show considerable flexibility. N-terminal helix is loosely packed and varies in orientation from one subunit to another (**Fig. 2B** and **Fig. S1**), affecting distances between K6 and K16 and other lysine residues. The orientations of K6 and K16 side chains also vary significantly. In consequence, the K6–K116 C_α_ - C_α_ inter-residue distance varies between subunits from about 20 to 27 Å, and 23 Å on average (**Table 1** and **Fig. 4B**). The side chain of K116, located on the β5-5′ loop, is protruding into the inner side of the pentamer (the water-filled vestibule – **Fig. 4C** and **Fig. S2**). Thus, a cross-link can be produced between K6 at the top of the vestibule and K166, which is accessible from the inner vestibule of the protein. Given that GlyBP is dynamic and some segments of its backbone have considerable flexibility, this contact can be easily made.

K16–K116 crosslinks are not observed although the average C_α_- C_α_ distance in the model is well within the crosslinker range (21 Å on average). Similarly, crosslinks between K6 (or K16) and K95, which is located close to K116 on the inner side of the protein, theoretically fulfill the C_α_- C_α_ distance condition but have not been identified. The absence of these crosslinks is consistent with the fact that in our model this crosslink is sterically obstructed - K95 is located underneath an overhang formed by two segments: Pro10-Arg27 and Tyr78-Ser92. It is thus unlikely that the linker could interact with both K95 and any of the N-terminal lysine residues located at the top of the pentamer over this tightly packed bulge on the inner vestibule’s surface (**Fig. 4C**: right panel). In addition, we expect that the mobility of the K95 side chain is limited by a salt bridge with Asp114 from the adjacent subunit and/or with the neighboring Asp91. These salt bridge interactions vary between subunits but we found them to form and persist in two subunits. Interestingly, we did not observe crosslinks between K95 and K116. These lysine residues are proximal with side-chains facing the inner surface of the protein and their C_α_- C_α_ distance fulfills the linker length condition in all subunits. Thus they should be accessible to the linker through the inner vestibule (**Fig. 4C**). However, an examination of subunit interfaces (**Fig. S2**) reveals that subunits are densely packed against each other and K95 and K116 of one subunit are separated by predominantly Asp114 from the neighboring subunit. That makes them unlikely to be accessible to the linker and explains the lack of crosslinks in that region of GlyBP.

The remaining four observed intra-subunit crosslinks involve K190 and K193 on β9, and K200 and K206 on loop C. This is also fully consistent with our model. All these lysine residues, along with K33, are the only lysine residues accessible to the cross-linker as they are located the outer, readily accessible, surface of the pentamer (it is reasonable to assume that the degree of mobility and vestibule penetration by the cross-linker is rather small). The distances from K6 or K16 to K33, K190, K193, K200, or K206 are far apart to accommodate crosslinking. In our model, access to K104, located at the subunit interface (deep towards the inner side, see **Fig. 4C**), is sterically blocked by the adjacent subunit. K143 could potentially be crosslinked with K190 or K193. However, it is not as exposed as K190/193 (on the outer surface, see **Fig. 4C**) and, being buried completely or mostly under the surface, it cannot be accessed by the linker.

The pairs K190/K193 and K200/K206 are proximal but the dynamic nature of GlyBP allows lysine residues to be crosslinked over distances considerably shorter than that predicted in a static modeling protein molecule. K193 was crosslinked with both K200 and K206 consistent with our homology model (**Fig. 4B**). K193 C_α_- C_α_ distance to K200 and K206 is in the range of 20 and 22Å, respectively (18–24 Å taking into account variations between subunits). The contacts may be additionally facilitated by flexibility of loop C, which harbors both K200 and K206. Loop C is critical for ligand binding in the entire Cys-loop receptor family and it is believed to undergo large conformational changes. In case of a large-amplitude away-from-the-interface movement of C loop these distances could be reduced further. Moreover, such a conformational change might possibly allow K190/K200 or K190/K206 crosslinks to be formed as well. It is however, at present uncertain which residues precisely are the C loop hinge points, and to what degree the loop motion can affect crosslinking. Based on our current model, crosslinks involving K190 and K200/K206 are not likely to form as the C_α_- C_α_ distances are larger than the length of the crosslinker; in two subunits the conformation of the C loop is such that K190/K200 C_α_- C_α_ distance is approximately 25Å, otherwise it ranges from 27 to 32Å.

Of note, we did not detect any crosslinks involving K33. This lysine residue is located on the α helix-β1 loop that was identified in our models as being one of the most flexible parts of the protein with its side chain exposed on the outer surface. Thus this accessible residue is within crosslinking distance to K206 (intrasubunit link) and also to K6/K16 of the counterclockwise neighbor (intersubunit link). However, K33 crosslinking would prevent trypsin cleavage after this site, as the enzyme will no longer cleave after K33 when covalent modification renders it unrecognizable as a substrate target to trypsin. Any crosslink containing a K33 linkage then becomes part of a bigger peptide that now includes N38, a known site of glycosylation (with glycosylation usually being heterogeneous in baculoviral expression studies). Differential glycosylation is expected to give rise to indeterminant masses, and tryptic peptide fragments (G34-D59) that include N38 have never been observed in either control (see **Fig. 1**) or DMS-treated samples. Peptides encompassing this site was also never observed in any of our MS studies of full-length GlyR [62], [63]. While we presume K33 is accessible and reactive with DMS, we did not detect any crosslinked peptides containing this site in these studies.

### Identification of intersubunit chemical crosslinks in GlyBP by MALDI-TOF MS

**Figure 5:**
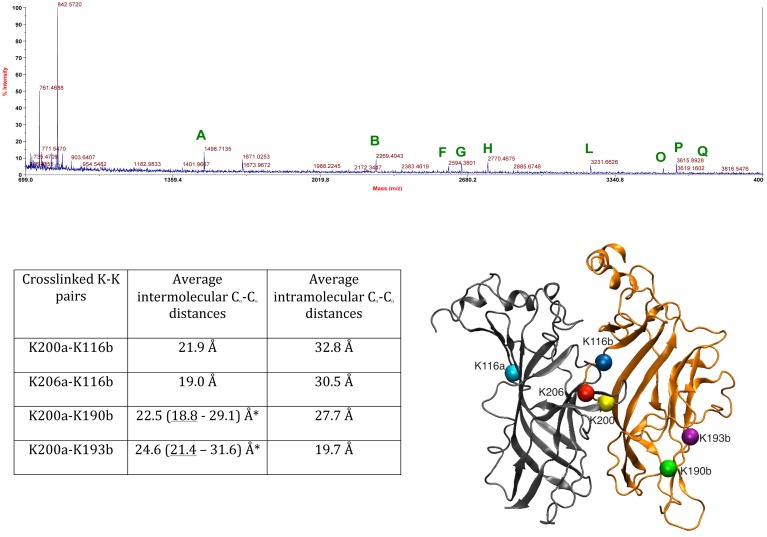
Intra-/inter-molecular crosslinks observed in GlyBP by MALDI-TOF MS analysis after crosslinking with DMS. Representative mass spectrum of tryptic digest of excised higher molecular weight GlyBP band is shown in the top panel. Mass peaks assigned as crosslinked peptides are labeled and further identified in **Table 2**. Average C_α_-C_α_ Lys-Lys distances measured along the MD trajectory of assigned crosslinks are provided in the panel below. The calculation of distances is averaged over all 5 subunits/interfaces over the 2 ns long MD trajectory. a and b indices distinguish adjacent GlyBP monomers in a pentamer. The positions of the Lys residues in two neighboring subunits of the GlyBP model are shown in bottom right. The protein structure is shown in grey and gold color in cartoon representation and C_α_ atoms of Lys residues are shown as colored spheres. * the range of distances reflect variations the average distance between subunits in the MD trajectory; while upper range distances are greater that the crosslinker length, the flexibility of the C loop in GlyBP brings the distances (underlined) well within the crosslinker arm length.

As described above, identified crosslinks present in lower-molecular weight bands from SDS-PAGE of purified GlyBP can only arise from intramolecular crosslinking events. Gel slices from oligomeric bands were also excised and subjected to similar analyses (see **Fig. 5A** for a representative MS spectrum). Any K-K crosslinks detected in higher order bands from SDS-PAGE that contain oligomeric GlyBP may be expected to result from either intra- (two crosslinked lysines were crosslinked within the same protein molecule) or inter-subunit crosslinks (two crosslinked lysines were from neighboring subunits of oligomeric GlyBP). As expected, many of the identified masses and deduced crosslinked peptides (**Table 2**) were identical to those found in monomeric GlyBP bands. However, several mass ions were uniquely observed only in the high-order GlyBP oligomeric bands (**Table 2** highlighted in red and blue). We hypothesize that these mass ions are the result of inter-molecular crosslinking events. The assigned K-K pairs were identified as K116b-K200a, K116b-K206a, K200a-K190b and K200a-K193b, where a and b denote two different neighboring subunits, counterclockwise looking down the pentamer axis from the N-terminus (highlighted in red; designations of a and b were predicted from our GlyBP model). Among these crosslinks, the masses 2652.193 (EEKDLR_191–196_-DLRYCTKHYNTGK_194–206_) and 3153.609 (FILKEEKDLR_187–196_-DLRYCTKHYNTGK_194–206_) are of particular interest since both masses were fit to crosslinked peptides that cannot arise intramolecularly, as they crosslink overlapping peptide sequences (highlighted in blue in **Table 2**). Both masses indicated that K200 was crosslinked to K193 in a neighboring subunit.

**Table 2:** Intra-/Intermolecular crosslinks identified by mass spectrometric studies of higher order oligomeric GlyBP bands.

K-K crosslinks	Cross-linked peptides	Theoretical m/z	Observed m/z	ΔMass, ppm*	N*	Peak*
190–193	187–196	1498.795	1498.743	−35	8	A
193–200	191–196, 197–206	2268.040	2268.105	−29	6	B
200–206	197–213	2357.048	2357.113	27	6	C
*190–200*	*187–193, 197–206*	*2385.159*	*2385.088*	*30*	*8*	*D*
N-6/16	1–2, 3–20	2430.195	2430.126	29	9	E
193–206	191–196, 201–213	2593.214	2593.177	15	7	F
***193–200***	***191–196, 194–206***	***2652.252***	***2652.193***	***22***	***6***	***G***
*190–200*	*187–193, 194–206*	*2769.371*	*2769.317*	*20*	*8*	*H*
193–206	191–200, 201–213	3145.452	3145.511	–19	9	I
***190/193–200***	***187–196, 194–206***	***3153.584***	***3153.609***	−***8***	***9***	***J***
193–200	191–200, 194–206	3204.489	3204.405	26	6	K
*116–200*	*105–119, 197–206*	*3230.549*	*3230.419*	*40*	*8*	*L*
*190–200/206*	*187–193, 197–213*	*3262.571*	*3262.502*	*21*	*7*	*M*
6–116	3–16, 105–119	3478.711	3478.634	22	6	N
*116–206*	*105–119, 201–213*	*3555.724*	*3555.670*	*15*	*9*	*O*
*116–200*	*105–119, 194–206*	*3614.761*	*3614.823*	−*17*	*8*	*P*
190/193–200/206	187–196, 197–213	3646.783	3646.656	35	7	Q
N/6–116	1–16, 105–119	3705.849	3705.907	−16	6	R

*the ΔMass is the maximum observed ppm difference between theoretical and observed m/z over N, the number of times this m/z peak was observed in 10 independent experiments. See **Fig. 5** for corresponding assigned peak in representative MALDI-TOF spectrum.

Unique bands found in higher order oligomeric GlyBP bands (absent in monomeric bands) and assigned as intermolecular crosslinks are *italicized*, with ***bold*** indicating assignments that ***cannot*** be assigned as an intramolecular crosslinks.

Importantly, all crosslinks uniquely identified only in extracts from oligomeric GlyBP are consistent with the homology model of GlyBP. The calculated distances for intersubunit K116–K200 and K116–K206 crosslinks fall within the range of possible crosslinking distance with DMS (19–22 Å). In contrast, our model showed that, for these two crosslinked pairs, intrasubunit crosslinking is not possible since the predicted intramolecular distances in the GlyBP model would be too large to be spanned by DMS (31–33 Å) (**Table 2** and **Fig. 5B**). In addition, such crosslinks would be energetically unfavorable as it would require the covalent crosslink to pass through the close-packed protein interior, while the intermolecular covalent linker traverses the solvent accessible surface. The other set of observed unique inter-subunit crosslinks identified in oligomeric bands: K200 with K190/K193 of the adjacent subunit, are fully consistent with our homology model. The trajectory average C_α_- C_α_ intersubunit distances between K200 and K190/193 are 23 and 25 Å (**Table 2**), respectively, but across some subunit interfaces these distances amount to 19 and 21 Å, thus falling well within the linker arm length.

## Discussion

Given the recent advances in MS technology and its exquisite sensitivity, MS has the potential to be a powerful structural tool. The approach explored in this study is to introduce chemical crosslinks of known length into a complex oligomeric protein and subsequently identify crosslinked residues. Experimentally deduced distance constraints can then be used in model building studies as an input to all atom MD simulations. While these distance constraints are not on the same scale as those determined from other high resolution methods such as x-ray crystallography and NMR, a network of systematically-generated crosslinks can critically evaluate structural models [27], [28], [32], [36], [51], [64], [65]. Importantly, this approach may be used under conditions that challenge or limit other biophysical methodologies. MS studies are not precluded by the presence of the bilayer, so they are easily adapted for studying membrane proteins. Chemical crosslinking studies may be conducted under physiological concentrations and conditions, and this approach may be used in studying large oligomeric complexes. In addition, the exquisite sensitivity of modern spectrometers allows one to examine low abundance proteins, such as membrane proteins.

While these methods could be used to examine full-length GlyR, we chose to instead examine GlyBP, a structural and functional homolog of the ECD of GlyR. GlyBP was used for ease of study, as we can express and purify mg quantities of this soluble homopentameric protein. Importantly, for MS studies of oligomeric assemblies, one needs to be able to distinguish between intra- and intersubunit crosslinks in order to provide useful information regarding distance constraints for model building/validation. In this study, we have hypothesized that any unique masses corresponding to crosslinked GlyBP peptides in oligomeric bands that are absent in monomeric bands may be attributed to crosslinks between neighboring subunits in the GlyBP pentamer. Thus, we have used our model of GlyBP to critically test this hypothesis as we are confident that our model is qualitatively correct given the wealth of biochemical data that the crystal structures of AChBP and the ECD of bacterial pLGICs are structural homologs of the ECD of all pLGICs [25], [66]–[68]. While these models may have limitations in accurately predicting sidechain placement, the backbone and broad details of these models, including our models of the ECD of GlyR [24] and of GlyBP, appear to be valid.

Though limited to DMS-induced Lys-Lys crosslinks, the identified intra- and intersubunit crosslinks are consistent with our initial model, and show proof of principle. The validation of this methodology allows one to confidently apply these methods in subsequent studies to further refine structural models using systematically introduced crosslinks of varying length (e.g. via introduced single site Cys residues and thiol-specific crosslinkers). Most importantly, these studies show the validity of the assumption that unique masses only identified in MS analyses of higher order bands are due to inter-subunit crosslinks. Given the ability to discriminate between inter-and intra-subunit crosslinks, these studies illustrate the capability of systematic comprehensive crosslinks to resolve structures at high resolution. Significantly, we show that single-dimensional MALDI-TOF MS studies of limited resolution are sufficient to identify crosslinked peptides in trypsinized samples of purified monomeric and oligomeric protein bands from SDS-polyacrylamide gels. Since this validated methodology may be amenable to a wide range of researchers with limited accessibility to high end MS platforms, broadly available MS platforms are capable of providing information useful in characterizing allosteric states of large complexes that are less amenable to NMR and crystallographic studies. In order to understand the functioning of complex allosteric machines in the membrane, such as pLGICs, novel methodologies must be developed that are capable in providing state-dependent information regarding the receptor in various forms (e.g., resting, open, or desensitized states in the case of pLGICs). Since crosslinking studies may be conducted in different liganded states (and hence, different allosteric states) of the receptor, these types of studies also have the potential to resolve structural changes involved in gating and desensitization. While the structural changes involved in channel gating and desensitization may be quite subtle, the introduction of systematically generated crosslinks with different lengths should be able to resolve small global changes.

Similar approaches are widely utilized in refining structures using NMR and x-ray crystallographic studies in which initial models of the protein and a set of experimentally-determined distance constraints allows one to revise one’s model and produce a revised conformation. The benefit of such approach is that one does not need to *a priori* predict precise conformational transitions nor relative rigidity (or deformability) of individual elements of protein structure. For such conformational refinement one also does not need a complete set of distance constraints. A few strategically and carefully chosen distance constraints is sufficient to predict the direction of the conformational transition of the protein regardless whether they are short- or long-distance constraints (the limitation being the precision, not the length, of the constraints). In MD simulations the elements of the protein structure have correct relative deformability properties as evidenced by extensive comparisons of MD simulations with NMR derived structural and relaxation properties, such as J-couplings and dipolar relaxation [69]. For example, the α-helices and individual β-sheets are relatively rigid, while the loops and turns are relatively flexible. Large β-structures however may undergo undulations of the surface that result in significant shifting of relative position of the sheet edges with respect to each other without visible local deformations or breaking of hydrogen bonds. Such changes can lead to a significantly altered conformation of the protein structure yet would be difficult to detect by methods that probe changes of small distances. We propose that large sets of systematically-generated crosslinks has the potential to detect and identify conformational rearrangements of the structure that are manifested by small, subtle changes in local distances.

## Supporting Information

Figure S1 Root mean squared fluctuation profiles for individual subunits and the pentamer average (thick read line) over the last 360 ps. The individual fluctuation profiles differ predominantly in the regions of the N-terminus and loops.
(TIF)

Figure S2 Space-filled model showing subunit interface packing. Lysine residues are shown as Van der Waals spheres: K95 - orange, K116 - cyan, and K6 - blue.
(TIF)
