# Supplementary figures and images for: Crosslinking Constraints and Computational Models as Complementary Tools in Modeling the Extracellular Domain of the Glycine Receptor
Source: PLoS One. 2014 Jul 15;9(7):e102571. doi: 10.1371/journal.pone.0102571 (PMC4099341; doi:10.1371/journal.pone.0102571)

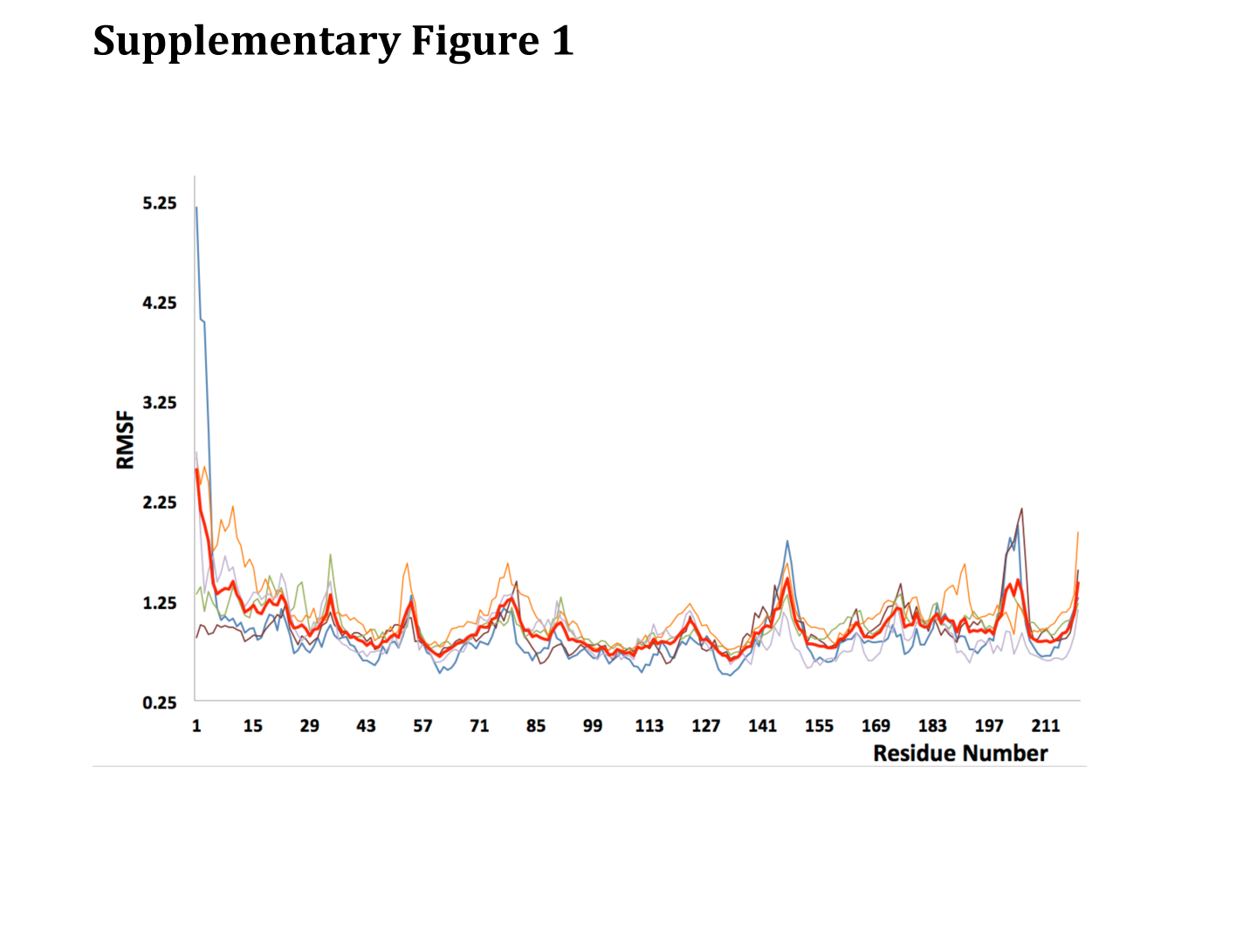

Supplement: Figure S1 — Root mean squared fluctuation profiles for individual subunits and the pentamer average (thick read line) over the last 360 ps. The individual fluctuation profiles differ predominantly in the regions of the N-terminus and loops. (TIF) [file pone.0102571.s001.tif]

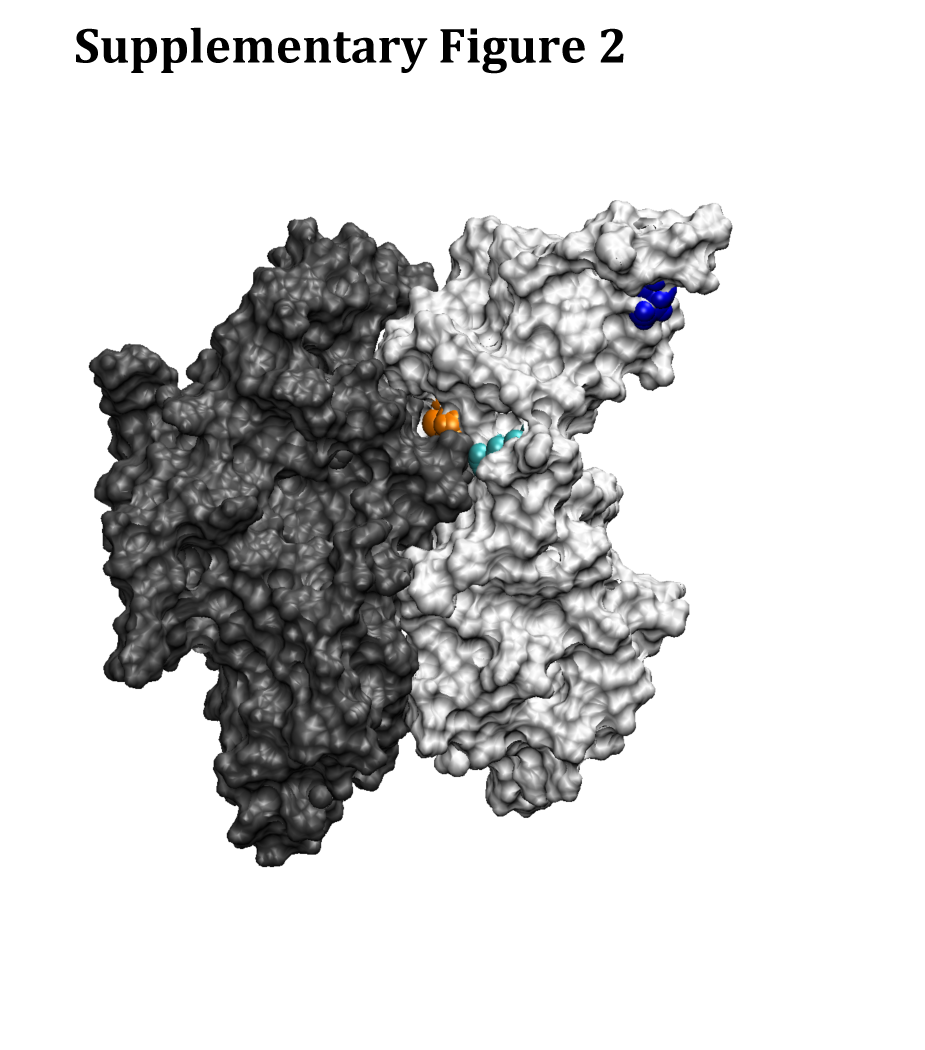

Supplement: Figure S2 — Space-filled model showing subunit interface packing. Lysine residues are shown as Van der Waals spheres: K95 - orange, K116 - cyan, and K6 - blue. (TIF) [file pone.0102571.s002.tif]
